# Scoping review of cytolytic vaginosis literature

**DOI:** 10.1371/journal.pone.0280954

**Published:** 2023-01-26

**Authors:** Roni Kraut, Fabiola Diaz Carvallo, Richard Golonka, Sandra M. Campbell, Anoush Rehmani, Oksana Babenko, Mao-Cheng Lee, Pedro Vieira-Baptista

**Affiliations:** 1 Department of Family Medicine, University of Alberta, Edmonton, Alberta, Canada; 2 School of Public Health, University of Alberta, Edmonton, Alberta, Canada; 3 John W. Scott Health Sciences Library, University of Alberta, Edmonton, Canada; 4 Faculty of Kinesiology, University of Calgary, Calgary, Alberta, Canada; 5 Department of Laboratory Medicine and Pathology, University of Alberta, Edmonton, Alberta, Canada; 6 *DynaLIFE* Medical Laboratories, Edmonton, Alberta, Canada; 7 Hospital Lusíadas Porto, Porto, Portugal; 8 Lower Genital Tract Unit, Centro Hospitalar de São João, Porto, Portugal; Al-Jouf University College of Medicine, SAUDI ARABIA

## Abstract

**Background:**

Cytolytic vaginosis (CV) is a little-known, controversial condition that is typically not considered for women presenting with vulvovaginitis symptoms. Objective: The objective of this scoping review was to identify and compile the global evidence on CV.

**Methods:**

A medical librarian searched Prospero, Wiley Cochrane Library, Ovid Embase, Ovid Medline, EBSCO CINAHL, ProQuest Dissertations and Theses Global, and Scopus, from inception to April 4, 2019 and updated to October 17, 2021. Studies were eligible if they discussed CV. Two independent reviewers conducted study selection and data extraction.

**Results:**

Sixty-four studies were identified, with 67% of studies (n = 43) published since 2007. Studies were from around the world, including the United States (28%, n = 18), Brazil (11%, n = 7), Portugal (11%, n = 7), and China (11%, n = 7). Fifty percent of studies (n = 32) were reviews; the remainder were observational; and of these, 78% (n = 25) were cross-sectional. The most frequent topics included: diagnosis (19%, n = 12), prevalence (17%, n = 11), and overview of CV (50%, n = 32). Evidence for prevalence in symptomatic women (median prevalence of 5%, interquartile range 3%-8%) was based only on 16% of studies (n = 10) with minimal evidence on prevalence in asymptomatic women and across different geographic regions. Microbiological findings, including abundant lactobacilli and fragmented epithelial cells, were found useful to distinguish between CV and vulvovaginal candidiasis, and *Lactobacillus crispatus* was noted to dominate the vaginal flora in women with CV. Most studies used subjective criteria to diagnose CV as the condition lacks gold-standard microscopic criteria. The suggested primary treatment (baking soda irrigations) was largely based on expert opinion, and there was minimal evidence on associations between CV and other conditions.

**Conclusion:**

Knowledge gaps currently exist in all realms of CV research. Additional research is needed to confirm the validity of CV and ensure that women are diagnosed and treated effectively.

## Introduction

Vaginitis has a high global prevalence and economic burden, and has a significant impact on woman’s physical health, mental health, and overall function [1–5]. The differential diagnosis for vaginitis is broad (S1 Table) and it remains controversial whether a dysbiosis pattern seen on wet mount called cytolytic vaginosis (CV) should be included in the differential diagnosis.

Evidence about CV appears in literature as early as 1961 [6], yet, it was not until 1991 that Leonard Cibley and Laurence Cibley coined the term CV after encountering women with symptoms similar to vulvovaginal candidiasis (white discharge, irritation, pruritus) but with a markedly different pathophysiology and treatment [7]. They published a narrative paper on CV; it described this entity, proposed diagnostic criteria, and described treatment. However, the paper did not provide any quantitative patient data including demographics, symptoms, diagnosis results, and treatment outcome. Since then, CV has remained a largely unknown, controversial, and understudied condition. It is still questioned whether it is an actual condition, with some asserting that the symptoms are physiological [8], and it is typically not listed as a condition in vaginitis guidelines [9].

A critical appraisal of CV was published in 2020 and examined whether CV should be seen as a true condition [10]. Appropriate to a critical appraisal, the authors examined evidence from published articles they were aware of (n = 10) and provided an opinion on the existence of CV. However, there has not yet been a scoping review of CV. A scoping review is different from both a critical appraisal and a systematic review; instead of answering a specific question, it seeks to delineate evidence available, identify knowledge gaps, define concepts, or examine research methodology [11].

Our objective was to complete a scoping review of CV to uncover all evidence and identify knowledge gaps related to the following aspects of CV: prevalence, diagnosis, treatment, and associations between CV and other conditions.

## Methods

We used the Preferred Reporting Items for Systematic Reviews and Meta-Analysis (PRISMA) guidelines for scoping reviews (S2 Table) for reporting this review [12]. A review protocol for this study does not exist.

### Search strategy

A medical librarian (S.C.) searched Prospero, Wiley Cochrane Library, Ovid Embase, Ovid Medline, EBSCO CINAHL, ProQuest Dissertations and Theses Global, and Scopus. All databases were searched from inception to April 4, 2019 and updated to October 17, 2021. The search strategy included both text words and controlled vocabulary (e. g., MeSH, EMTREE) for the concepts “*Lactobacillus*,” “cytolysis,” and “vaginal.” No limits were applied. Results (219 studies) were exported to COVIDENCE citation management software, where duplicates (116 studies) were identified and removed. Search strategies and the PRISMA-S checklist are included in S1 File and S3 Table, respectively.

### Study selection

Studies were included if they discussed CV. Studies did not need to use the term “cytolytic vaginosis,” but they needed to indicate that the condition involved excess lactobacilli and cytolysis. Studies also needed to have a discussion on CV, however brief, to be included.

Two independent reviewers (R.K. and F.D.C.) first screened the abstract and the title of studies for eligibility and then reviewed the full text of the eligible studies to determine whether they met the inclusion criteria. References of the included studies that focused on CV were reviewed. Studies in other languages were translated into English with either Google Translate or an online translating service. Excel spreadsheets were used to track the selection of studies. Any disagreements were resolved with discussion, and we calculated the percentage of agreement between study selectors.

### Data extraction

The data extracted from all relevant studies included:

Descriptive data: (1) year published; (2) form of publication: journal, abstract, presentation, book, and chapter/section of book; (3) study location (if the study did not provide the location, the location of the authors was used as the study location); (4) language; (5) funding source; (6) journal title; (7) journal impact factor (2018 InCites Journal Citation Report); (8) type of study: review, case report, case series, cross-sectional descriptive, cross-sectional analytical, case control, prospective cohort, retrospective cohort, and randomized controlled trial; (9) CV focus of study: yes or no; (10) aspect of CV: prevalence, diagnosis, treatment, associations, comprehensive; (11) number of women; and (12) study objective.

Prevalence data: (1) participant selection: location of recruitment, date of recruitment, exclusion criteria, and sampling method; (2) participant age; (3) vulvovaginal symptoms: yes or did not indicate; (4) negative yeast microscopy; (5) negative yeast culture; and (6) number of women with CV (in total and in a subgroup).

Diagnostic data: (1) location of sample; (2) swab type; (3) stain used; (4) CV diagnosis criteria: Cibley criteria or other criteria; (5) description/criteria for lactobacilli; (6) description/criteria for cytolysis; and (7) vaginal flora classification system.

Treatment: (1) primary or secondary source; (2) recommended treatment: baking soda sitz bath, baking soda vaginal irrigations, tampons, antibiotics, other treatment, and order of treatment; and (3) results of treatment.

Association: (1) exposure/outcome; (2) selection bias based on National Institutes of Health (NIH) quality assessment tools [13]; (3) information bias based on NIH quality assessment tools [13]; (4) confounder bias based on NIH quality assessment tools [13]; and (5) study results. Bias was assessed for studies with a focus on complication to help determine the credibility of the results.

Data were extracted by at least two independent reviewers, including R.K., F.D.C., R.G., A.R., and O.B. Reviewers first calibrated data on the first few studies together and then extracted data independently. Disagreements were resolved through discussion, and we calculated the percentage agreement between data extractors.

### Synthesis of results

The descriptive data, diagnosis data, and treatment data were analyzed in Excel and summarized in figures organized by attribute. The median prevalence was calculated by subgroup and plotted using a forest plot. Studies specific to diagnosis were organized by focus and shown in a table. Studies on associations between CV and other conditions were organized by type and listed in a table. The data synthesis was done in Word and Excel.

## Results

### Study selection and data extraction

**Fig 1:**
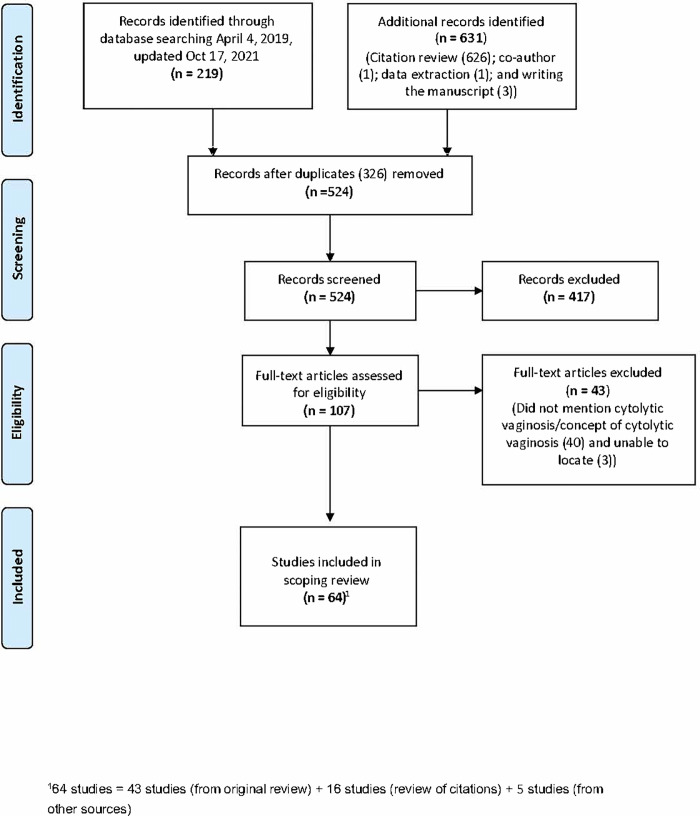
PRISMA flow diagram.

The total number of unique studies found was 524; of these, 64 met the selection criteria (43 from the original search, 16 from the reference search, and 5 from other sources Fig 1). The percentage of agreement between reviewers was 84% on study selection and 86% on data extraction. Table 1 lists the characteristics of the included studies, and the full data set is included in S4 Table.

**Table 1:** Characteristics of studies in the scoping review (n = 64 studies).

Publication	Location	Language	Type	CV focus	Aspect	Total women	Objective
ACOG Practice Bulletin N. 72. 2006 [14]	United States	English	practice guideline	N	comprehensive	NA	Approach to vaginitis
Akgun 2012 [15]	Turkey	English	retrospective cohort	Y	association	4672	Relationship between CV and infertility
Amaral 2007 [16]	Brazil	English	cross-sectional analytical	N	prevalence	155	Prevalence of abnormal flora and association between douching and abnormal flora
Anderson 2016 [17]	United States	English	review	Y	comprehensive	NA	Overview of CV
Andrist 2001 [18]	United States	English	review	N	comprehensive	NA	Approach to vaginal infections
Azevedo 2019 [19]	Portugal	English	cross-sectional analytical	N	diagnosis	50	Impact vaginal sampling site has on wet mount microscopy results
Batashki 2009 [20]	Bulgaria	Bulgarian	cross-sectional descriptive	Y	prevalence	1152	Prevalence of CV in women with vulvovaginal symptoms
Beghini 2015 [21]	Brazil	English	cross-sectional analytical	N	diagnosis	209	Comparison of metabolites (D-lactic acid, L-lactic acid, EMMPRIN and MMP-8) in vaginal samples of women with vaginosis
Bibbo 1988 [22]	United States	English	cross-sectional descriptive	N	prevalence	15000	Prevalence of abnormal microbiology in outpatient Pap samples
Cerikcioglu 2004 [23]	Turkey	English	cross-sectional descriptive	Y	prevalence	210	Prevalence of CV in women with signs/symptoms of vulvovaginal candidiasis
Cibley 1991 [7]	United States	English	review	Y	comprehensive	NA	Overview of CV
Demirezen 2003 [24]	Turkey	English	cross-sectional descriptive	Y	prevalence	2947	Prevalence of CV in women with symptoms of vulvovaginal candidiasis
Donders 1999 [25]	Belgium	English	review	N	diagnosis	NA	Classification of vaginal flora into grades based on quantity of lactobacilli
Donders 2007 [26]	Belgium	English	review	N	comprehensive	NA	Diagnostic techniques for abnormal vaginal flora and characterization of abnormal vaginal flora
Donders 2010 [27]	Belgium	English	review	N	comprehensive	NA	Management of recurrent vulvovaginal candidiasis
Edwards 2004 [28]	United States	English	review	N	comprehensive	NA	Approach to infectious vaginitis
Fan 2010 [29]	China	Chinese Mandarin	cross-sectional descriptive	N	prevalence	516	Characterization of clinical and microbiological findings of aerobic vaginitis
Faro 2004 [30]	United States	English	review	Y	comprehensive	NA	Overview of CV
Fontan 2020 [31]	Spain	English	cross-sectional analytical	Y	diagnosis	38	Evaluation of Lactobacillus crispatus as a marker of CV
Gaspar 2019 [32]	Portugal	English	cross-sectional descriptive	N	diagnosis	24	Role of Lactobacillus crispatus in vaginal infections
Giraldo 2005 [33]	Brazil	Portuguese	cross-sectional analytical	N	prevalence	97	Impact vaginal intercourse and douching has on vaginal microbiota
Guevara 2011 [34]	Venezuela	Spanish	review	Y	comprehensive	NA	Overview of CV
Gupta 2019 [35]	India	English	review	N	comprehensive	NA	Analysis of the impact vaginal microbiome has on female health
Hacisalihoglu 2021 [36]	Turkey	English	cross-sectional descriptive	Y	comprehensive	2932	Prevalence, diagnosis, and treatment of CV
Hay 2018 [37]	United States	English	review	N	comprehensive	NA	Approach to vaginal discharge
Hills 2007 [38]	United States	English	review	Y	comprehensive	NA	Overview of CV and lactobacillosis
Hu 2015 [39]	China	English	cross-sectional descriptive	Y	diagnosis	108	Comparison of microbiological characteristics of CV and vulvovaginal candidiasis
Hutti 2000 [40]	United States	English	review	Y	comprehensive	NA	Overview of CV
Jiang 2012 [41]	China	Chinese Mandarin	cross-sectional descriptive	N	prevalence	1260	Prevalence of different types of vaginal infections in women attending an outpatient clinic
Kaufman 1989 [8]	United States	English	review	N	comprehensive	NA	Overview of miscellaneous vaginal disorders
Korenek 2003 [42]	United States	English	review	Y	comprehensive	NA	Overview of bacterial vaginosis, lactobacillosis, and CV
Lapina 2020 [43]	Russia	Russian	prospective cohort	N	treatment	60	Impact of Zalain (vaginal suppositories) with Zalagel Intim (gel) post genital prolapse surgery on risk of vaginal dysbiosis
Ledger 2017 [44]	United States	English	review	Y	comprehensive	NA	Overview of CV, aerobic vaginitis, and desquamative inflammatory vaginitis
MartinSaco 2019 [45]	Spain	English	review	N	comprehensive	NA	Evidence review of lesser known vaginitis conditions
Mills 2017 [46]	United States	English	review	N	comprehensive	NA	Approach to vaginitis
Moghaddam 2009 [47]	Iran	English	case control	N	association	415	Association between types of Lactobacilli flora and vulvovaginal candidiasis
Mulley 2000 [48]	United States	English	review	N	comprehensive	NA	Approach to vaginal discharge
Nasiell 1972 [49]	Sweden	English	cross-sectional analytical	N	association	440	Association between lactobacilli/CV and cervical dysplasia/cervical cancer
Paavonen 1995 [50]	Finland	English	review	N	comprehensive	NA	Approach to vulvodynia
Puri 2019 [51]	India	English	cross-sectional descriptive	Y	prevalence	190	Prevalence of CV
Ramirez-Santos 2008 [52]	Spain	English	review	N	comprehensive	NA	Approach to recurrent vulvovaginitis
Raykova 2018 [53]	Bulgaria	English	cross-sectional descriptive	Y	prevalence	468	Prevalence of CV compared to vulvovaginal candidiasis and bacterial vaginosis in women presenting with vaginal discharge
Ricci 2010 [54]	Chili	Spanish	review	Y	comprehensive	NA	Overview of CV
Rocchetti 2011 [55]	Brazil	English	cross-sectional analytical	N	association	405	Prevalence and risk factors of group B streptococci in pregnant women
Sanches 2018 [56]	Brazil	English	cross-sectional analytical	Y	diagnosis	24	Characterization of the vaginal lipids concentration in vaginal discharge of vulvovaginal candidiasis, CV and normal vaginal flora samples
Sanches 2020 [57]	Brazil	English	cross-sectional analytical	Y	diagnosis	24	Clinical and laboratory characteristics to differentiate CV and vulvovaginal candidiasis
Secor 1992 [58]	United States	English	review	Y	comprehensive	NA	Overview of CV
Shopova 2001 [59]	Bulgaria	Bulgarian	review	N	comprehensive	NA	Overview of clinical and microbiological characteristics of lactobacillus
Shopova 2006 [60]	Bulgaria	Bulgarian	case series	Y	comprehensive	47	Case series of women with CV
Silva 2014 [61]	Brazil	English	retrospective cohort	N	association	3390	Factors associated with evolution of cervical intraepithelial lesions
Suresh 2009 [62]	India	English	review	Y	comprehensive	NA	Overview of CV
Vertolini 2014 [63]	United States	English	review	N	comprehensive	NA	Review of lactobacillosis
Vieira-Baptista 2017 [64]	Portugal	English	case control	N	association	291	Association between vaginal flora and vulvodynia
Vieira-Baptista 2017 [65]	Portugal	English	cross-sectional analytical	Y	association	1022	Association between CV and cervical dysplasia/HPV
Vieira-Baptista 2019 [66]	Portugal	English	review	N	comprehensive	NA	Overview of vaginitis
Vieira-Baptista 2020 [67]	Portugal	English	review	N	comprehensive	NA	Critical analysis of current diagnostic approach for vaginitis
Vieira-Baptista 2021 [68]	Portugal	English	review	N	diagnosis	NA	Establish evidence-based recommendations for wet mount microscopy
Voytik 2020 [10]	United States	English	review	Y	comprehensive	NA	Evidence appraisal of CV
Wathne 1994 [69]	Sweden	English	cross-sectional descriptive	N	prevalence	101	Comparison of clinical and microbiological findings in women with vaginal discharge
Xiao 2010 [70]	China	Chinese Mandarin	review	Y	comprehensive	NA	Overview of CV
Xu 2019 [71]	China	English	cross-sectional analytical	Y	diagnosis	75	Characterization of microbiome of women with CV with high-throughput sequencing
Yang 2017 [72]	China	English	cross-sectional descriptive	Y	diagnosis	536	Clinical differences between CV and vulvovaginal candidiasis in women with recurring vulvovaginitis
Yang 2020 [73]	China	English	cross sectional analytical	Y	diagnosis	149	Microbial composition and variation in lactobacillus microbiome in patients with CV compared to healthy controls
Zidovsky 1963 [74]	Czech Republic	English	retrospective cohort	Y	association	953	Association between CV and fetal impairment

### Descriptive statistics

Table 2 provides a summary of the descriptive statistics.

**Table 2:** Summary of descriptive statistics (n = 64 studies).

		Study reference numbers	Number of studies	Percentage of studies^e^
**Date of publication^a^**				
	1963–1991	7–8, 22, 49, 74	5	8%
	1992–2006	14, 18, 23–25, 28, 30, 33, 40, 42, 48, 50, 58–60, 69	16	25%
	2007+	10, 15–17, 19–21, 26, 27, 29, 31, 32, 34–39, 41, 43–47, 51–57, 61–68, 70–73	43	67%
**Continent** ^**b**^				
	Europe	15, 19, 20, 23–27, 31, 32, 36, 43, 45, 49, 50, 52, 53, 59, 60, 64–69, 74	26	41%
	Asia	29, 35, 39, 41, 47, 51, 62, 70–73	11	17%
	North America	7, 8, 10, 14, 17, 18, 22, 28, 30, 37, 38, 40, 42, 44, 46, 48, 58, 63	18	28%
	South America	16, 21, 33, 34, 54–57, 61	9	14%
**Type of publication**				
	Journal article	7, 10, 14, 16–21, 23–29, 33–43, 45–47, 49–63, 67–74	53	83%
	Book section/chapter	8, 22, 30, 44, 48, 66	6	9%
	Abstract	15, 31, 32, 64, 65	5	8%
**Language**				
	English	7, 8, 10, 14–19, 21–28, 30–32, 35–40, 42, 44–53, 55–58, 61–69, 71–74	54	84%
	Mandarin	29, 41, 70	3	5%
	Bulgarian	20, 59, 60	3	5%
	Spanish	34, 54	2	3%
	Russian	43	1	2%
	Portuguese	33	1	2%
**Funding** ^**c**^				
	From foundation	21, 55–57, 71, 72	6	10%
	From medical university	53	1	2%
	No funding	36, 61, 62, 67	4	7%
	Not mentioned	7, 10, 14, 16–20, 23–29, 33–35, 37–43, 45–47, 49–52, 54, 58–60, 63, 68–70, 73, 74	42	72%
	Unable to determine (abstract)	15, 31, 32, 64, 65	5	9%
**Type of journal** ^**d**^				
	Obstetrics/gynecology	7, 14, 16, 19–21, 23, 25–27, 29, 32–34, 39, 43, 45, 46, 50, 54, 55, 57, 59, 60, 62–65, 68–70, 72, 73	33	58%
	General medical	37, 47, 56, 61	4	7%
	Nurse practitioner	17, 38, 40, 58	4	7%
	Microbiology	35, 41, 53, 71	4	7%
	Cytology	36, 49, 51, 74	4	7%
	Dermatology	28, 52	2	4%
	Infectious disease/sexually transmitted infections	10, 67	2	4%
	Nursing	18, 42	2	4%
	Pathology	15	1	2%
	Public health	24	1	2%
**Journal impact factor** ^**d**^				
	No impact factor	20, 23, 25, 29, 33, 34, 38, 41–43, 45, 47, 51–54, 59, 60, 62, 63, 67	21	37%
	<1	17, 24, 36, 57, 58	5	9%
	1–1.9	16, 18, 19, 27, 28, 32, 37, 39, 40, 46, 49, 61, 64, 68, 70–74	20	35%
	2.0–2.9	10, 15, 26, 35, 50, 55, 56,69	8	14%
	>4.9	7, 14, 21	3	5%
**Study design**				
	Review	7, 8, 10, 14, 17, 18, 25–28, 30, 34, 35, 37, 38, 40, 42, 44–46, 48, 50, 52, 54, 58, 59, 62, 63, 66–68, 70	32	50%
	Cross-sectional descriptive	20, 22–24, 29, 32, 36, 39, 41, 51, 53, 69,72	13	20%
	Cross-sectional analytical	16, 19, 21, 31, 33, 49, 55–57, 65, 71, 73	12	19%
	Prospective/retrospective cohort	15, 43, 61, 74	4	6%
	Case-control	47, 64	2	3%
	Case series	60	1	2%
**Focus on CV**				
	Yes	7, 10, 15, 17, 20, 23, 24, 30, 31, 34, 36, 38–40, 42, 44, 51, 53, 54, 56–58, 60, 62, 65,70–74	30	47%
	No	8, 14, 16, 18, 19, 21, 22, 25–29, 32, 33, 35, 37, 41, 43, 45–50, 52, 55, 59, 61, 63, 64, 66–69	34	53%
**Topic**				
	Comprehensive	7, 8, 10, 14, 17, 18, 26–28, 30, 34–38, 40, 42, 44–46, 48, 50, 52, 54, 58–60, 62, 63, 66, 67, 70	32	50%
	Diagnosis	19, 21, 25, 31, 32, 39, 56, 57, 68, 71–73	12	19%
	Prevalence	16, 20, 22–24, 29, 33, 41, 51, 53, 69	11	17%
	Associations	15, 47, 49, 55, 61, 64, 65,74	8	13%
	Treatment	43	1	2%

^a^ The cut-off for the first group is based on the year of Cibley et al.’s study [7] on CV; the cut-off for the subsequent groups was chosen to divide the remaining years equally between groups.

^b^ Europe: Belgium (3), Bulgaria (4), Czech Republic (1), Finland (1), Portugal (7), Russia (1), Spain (3), Sweden (2), Turkey (4); North America: The United States (18); South America: Brazil (7), Chili (1), Venezuela (1); Asia: China (7), India (3), Iran (1).

^c^ Total studies in this section is 58, rather than 64, as 6 were book chapters/sections.

^d^ Total studies in this section is 57, rather than 64, as 6 were book chapters/sections and 1 was a conference abstract not published in a journal.

^e^ The percentages may not total 100% due to rounding.

The studies were published between 1963 and 2021, with 67% of studies (n = 43) published since 2007. Studies were predominantly published in the United States (28%, n = 18); Brazil (11%, n = 7); Portugal (11%, n = 7); and China (11%, n = 7). Eighty-three percent (n = 53) were journal articles; 9% (n = 6) were sections or chapters in books; and 8% (n = 5) were abstracts. Eighty-four percent of publications were in English (n = 54); the remaining were in Mandarin (5%, n = 3); Bulgarian (5%, n = 3); Spanish (3%, n = 2); Russian (2%, n = 1); and Portuguese (2%, n = 1). Seventy-two percent of articles (n = 42) did not mention whether they had received funding; 7% (n = 4) of articles indicated they did not receive funding; and 12% of articles (n = 7) received funding from a foundation (n = 6) or a university (n = 1).

The articles were published in a broad array of journals, including obstetrics/gynecology (58%, n = 33); general medical journals (7%, n = 4); and nurse practitioner journals (7%, n = 4). Thirty-seven percent of articles (n = 21) were published in journals without an impact factor, and of the journals with an impact factor, the median impact factor was 1.5.

The study types included: reviews (50%, n = 32); cross-sectional descriptive (20%, n = 13); cross-sectional analytical (19%, n = 12); prospective and retrospective cohort (6%, n = 4); case control (3%, n = 2); and case series (2%, n = 1). CV was the focus of 47% of studies (n = 30); the remaining studies mentioned CV, but it was not their main focus. Studies examined various aspects of CV, including: diagnosis (19%, n = 12); prevalence (17%, n = 11); associations between CV and other conditions (13%, n = 8); and treatment (2%, n = 1). The remaining studies (50%, n = 32) provided an overview of CV, and this included reviews (n = 30), cross-sectional descriptive (n = 1), and case series (n = 1).

### Prevalence

**Fig 2:**
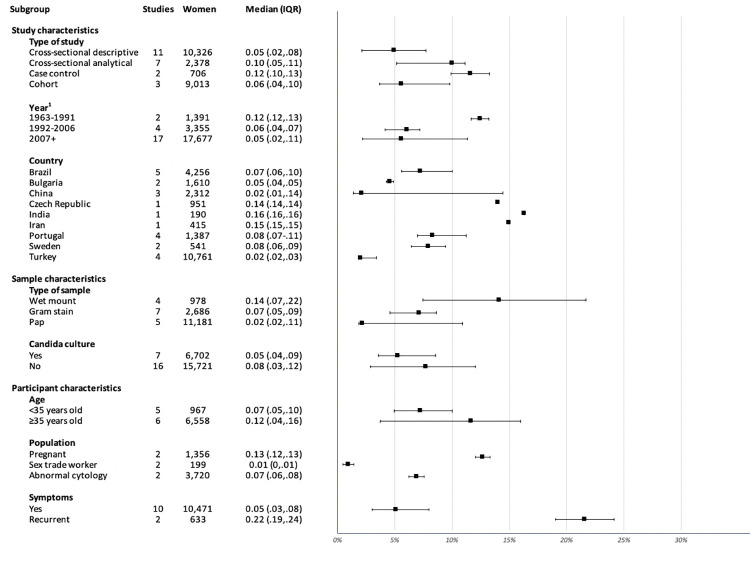
Median prevalence by subgroup. ^1.^The cut-off of the first group is based on the year of Cibley e’ al.’s study [7] on CV, and the cut-off for the subsequent groups was chosen to divide the remaining years equally between groups.

Twenty-three of the 32 non-review studies (72%) provided sufficient information to enable us to calculate the prevalence of CV for the study; Fig 2 shows the median prevalence by subgroup.

Cross-sectional descriptive studies had a lower median prevalence compared to the other study types. The median prevalence values in the Czech Republic, India, and Iran were outliers, likely because their prevalence was only based on one study and the study focused on a subpopulation. Studies using wet mount to diagnose CV had the highest median prevalence and the widest range, while studies using the Pap test had the lowest median prevalence. The studies using the Pap test did not indicate whether the conventional Pap approach or liquid-based cytology (less sensitive for flora evaluation) was used, although two of these studies were done after liquid-based cytology became available. In addition, performing a Candida culture appeared to have little impact on reported prevalence, and pregnant women and women with recurrent symptoms had a higher median prevalence.

### Diagnosis

Twenty-two of the 32 non-review studies (69%) provided diagnostic criteria. Table 3 summarizes the diagnostic criteria used in the studies.

**Table 3:** Summary of diagnosis variables (n = 22 studies).

		Study reference numbers	Number of studies	Percentage of studies^a^
Sample location				
	Vaginal, did not indicate specific location	24, 31, 39, 56, 57, 60	6	27%
	Did not indicate location	20, 49, 51, 53, 72, 74	6	27%
	Vaginal, lateral wall	16, 21, 33	3	14%
	Vaginal, multiple locations	19, 23, 47	3	14%
	Vaginal, posterior fornix	69, 73	2	9%
	Vaginal, front third of wall	29	1	5%
	Cervix	36	1	5%
**Swab type**				
	Did not indicate	20, 29, 31, 36, 47, 49, 51, 53, 60, 69, 74	11	50%
	Sterile cotton	21, 23, 39, 72, 73	5	23%
	Sterile dacron	16, 33, 56, 57	4	18%
	Endobrush	19	1	5%
	Wooden spatula	24	1	5%
**Stain used**				
	Gram stain	16, 20, 21, 23, 31, 33, 39, 53, 56, 60	10	45%
	Wet mount	19, 69	2	9%
	Gram stain and wet mount	29, 57, 72, 73	4	18%
	Papanicolaou	24, 36, 49, 51	4	18%
	Methenamine silver	47	1	5%
	Did not indicate	74	1	5%
**Criteria used**				
	Cibley criteria or variant of Cibley criteria	20, 21, 29, 36, 39, 51, 53, 56, 60, 69, 72, 73	12	55%
	Primarily lactobacilli and fragmented epithelial cells	16, 19, 23, 24, 31, 33, 47, 49, 74	9	41%
	Did not indicate	57	1	5%
**Classification of lactobacilli**				
	Subjective	16, 19, 20, 21, 23, 24, 29, 31, 33, 47, 49, 51, 53, 56, 60, 69, 72–74	19	86%
	Objective score	36, 39	2	9%
	Did not indicate	57	1	5%
**Classification of fragmented cells**				
	Subjective	16, 19, 20, 21, 23, 24, 29, 31, 33, 47, 49, 51, 53, 56, 60, 69, 72,-74	19	86%
	Objective	36, 39	2	9%
	Did not indicate	57	1	5%
**Vaginal flora grading system**				
	No grading system	20, 21, 23, 24, 29, 31, 36, 39, 51, 53, 56, 60, 69, 72–74	16	73%
	Grading system, no inclusion of CV as a grade	16, 19, 33, 49, 57	5	23%
	Grading system, inclusion of CV as a grade	47	1	5%

^a^ The percentages may not total 100% due to rounding.

Nine of these studies (41%) focused primarily on microbiology results for diagnosis (lactobacilli and cytolysis), while the other 12 studies (55%) used Cibley criteria or a variant of Cibley criteria, and 1 study (5%) did not indicate. Two studies (9%) provided scores/observations for diagnosing CV. Specifically, Hu et al. [39] was based on quantity of lactobacilli and cytolysis; Hacisalihoglu et al. [36] evaluated lactobacilli, neutrophils, cytolysis, *Candida* ssp. hyphae, and *Trichomonas vaginalis* on a scale of 0–3, based on number of colonies per oil immersion field. The remainder of the studies used subjective terms to characterize lactobacilli and cytolysis; for instance, “massive,” “abundant,” and “increased” for lactobacilli and “crowded with cellular debris,” “free nuclei,” and “marked” for cytolysis.

Sanches 2020 [57] and Shopova 2006 [60] used the Nugent score (gold standard for bacterial vaginosis); as a component of their diagnosis, both studies reported CV samples had a Nugent score of nil. Five studies classified vaginal flora into grades, and only Moghaddam et al.’s study [47] included a grade specific for CV with both lactobacilli and cytolysis.

In addition, there were 12 (19%) studies specifically focused on the topic of diagnosis (Table 4). Five of these studies examined how to distinguish between vulvovaginal candidiasis and CV and found that microbiological features appear to be more effective than clinical features. Four studies (6%) focused on *Lactobacillus* species and found that *Lactobacillus crispatus* dominates in women with CV; women with CV have less diverse lactobacilli microbiome; and *Lactobacillus crispatus* in women with CV secretes more acid. Two studies focused on wet mount findings for vaginal flora; the earlier study divided vaginal flora into grades based on lactobacilli and described CV as a variant of grade 1 (normal flora); and the later study provided evidence-based guidelines for vaginal wet mount microscopy. The remaining study found that an anterior fornix sample was more sensitive for CV.

**Table 4:** Diagnosis studies (n = 12 studies).

Publication	Location	Total women	Objective	Findings specific to CV
**Differences between CV and vulvovaginal candidiasis**				
Beghini 2015 [21]	Brazil	209	Comparison of metabolites (D-lactic acid, L-lactic acid, EMMPRIN and MMP-8) in vaginal samples of women with vulvovaginal candidiasis, CV, bacterial vaginosis, and normal flora	In CV, only L-lactic acid levels were significantly elevated compared to normal flora group.
Hu 2015 [39]	China	108	Comparison of microbiological characteristics of CV and vulvovaginal candidiasis	CV and vulvovaginal candidiasis can be differentiated based on quantity of *Lactobacillus* (CV > 1000 per OIF), fragmented epithelial cells, whole epithelial cells, and candida species.
Sanches 2018 [56]	Brazil	24	Characterize vaginal lipids concentration in vaginal discharge of women with vulvovaginal candidiasis, CV, and normal flora group	CV and vulvovaginal candidiasis have distinct lipid profiles. In CV, there were higher concentrations of lipids related to cellular apoptosis, oxidated stress, and bacterial overgrowth. In vulvovaginal candidiasis, there were higher concentrations of lipids related to inflammation and oxidative stress.
Sanches 2020 [57]	Brazil	24	Clinical and laboratory characteristics to differentiate CV and vulvovaginal candidiasis	The statistically significant differences between CV and vulvovaginal candidiasis were vaginal hyperemia, quantity of *Lactobacillus*, vaginal epithelium lysis, inflammatory process, pH, and Nugent score. The study did not look at quantity of vaginal discharge and timing of symptoms.
Yang 2017 [72]	China	536	Clinical differences between CV and vulvovaginal candidiasis in women with recurring vulvovaginitis	Statistically significant clinical differences between CV and vulvovaginal candidiasis including 1) less swelling, erosions, and ulcerations in women with CV; 2) increased quantity of discharge and discharge described as more paste-like in women with CV; and 3) symptoms primarily during ovulation and the luteal phase of the menstrual cycle with CV compared to being more evenly spread out with vulvovaginal candidiasis. We calculated likelihood ratios (S5 Table), and none of the 3 symptoms/signs are individually useful for diagnosis.
**Microbial composition**				
Fontan 2020 [31]	Spain	38	Whether *Lactobacillus crispatus* can be used as a marker for CV	*L*. *crispatus* prevalence in the CV group was 73.3% and 16.6% in the "normal microbiota" group.
Gaspar 2019 [32]	Portugal	24	Role of *L*. *crispatus* in vaginal infections	*L*. *crispatus* was dominant in women with CV and not dominant in women with vulvovaginal candidiasis.
Xu 2019 [71]	China	75	Use high-throughput sequencing to identify biomarkers for CV	1) There was increased microbial diversity in the normal flora group; 2) The density of *Lactobacillus* colonies was higher in CV group compared to normal flora group; 3) In CV, *L*. *crispatus* made up 97.5% of *Lactobacillus* species compared to 40% in the normal flora group.
Yang 2020 [73]	China	149	Microbial composition and variation in *Lactobacillus* microbiome in patients with CV compared to healthy controls	1) CV group had a less diverse *Lactobacillus* species; 2) *L*. *crispatus* had a higher prevalence in the CV group than the healthy flora group (88.7 vs. 56.4%); and 3) *L*. *crispatus* from the CV group produced more acid than *L*. *crispatus* from the healthy flora group.
**Other**				
Azevedo 2019 [19]	Portugal	50	Impact of sampling site on wet mount microscopy results	In CV, there was a higher sensitivity rate for anterior fornix samples; however, this was not statistically significant.
Donders 1999 [25]	Belgium	NA	Classification for Lactobacilli and defining the term aerobic vaginitis	Lactobacilli Grade 1 normal. Lactobacilli of variable sizes predominate. Grade 11 intermediate flora. Grade 11a lactobacilli still outnumber the other bacteria; 11b lactobacilli are less abundant than the other bacteria. Grade 111 complete disruption of normal lactobacilli. Includes CV as a variant of Grade 1 and describes wet mount findings of numerous lactobacilli, bare nuclei, and debris of cellular cytoplasm.
Vieira-Baptista 2021 [68]	Portugal	NA	Establish evidence-based recommendations for wet mount microscopy	CV can be distinguished on wet mount microscopy based on lactobacilli: abundant; leukocytes: absent; epithelial cells: variable degrees of cellular lysis (presence of bare nuclei and cytoplasm debris); background flora: only lactobacilli. And suggest avoiding examination immediately following menses.

### Treatment

Four of the 32 non-review studies (13%) provided results of treatment: Cerikcioglu et al. reported baking soda irrigation was effective in two women who used it [23]; Shopova et al. stated that 32 out of 47 women improved after baking soda irrigation [59]; Hacisalihoglu et al. reported that >95% of dyspareunia, discharge, and severe discomfort symptoms improved after a 10-day course of baking soda sitz baths [36]; and Lapina et al. treated women post cystocele surgery with sertaconazol vaginal suppository and an alkaline (pH 8–9) vaginal moisturizer and found that women with CV had 100% resolution of their symptoms [43]. Only Lapina et al. did further testing after symptomatic resolution, using pH, and found that pH normalized in women after the treatment [43].

We also looked at treatment recommendations among all studies due to the limited information from non-review studies. This is summarized in Table 5. Thirty-five (55%) studies mentioned treatment for CV, and 25 of these studies were review studies. The primary treatment recommended was baking soda; reasons centered around increasing the vaginal pH and decreasing the quantity of lactobacilli. Six of the 35 studies (17%) suggested discontinuing tampon use with the rationale that the menstrual flow is basic, raises the vaginal pH, and in doing so inhibits the growth of lactobacilli. Five of the 35 studies (14%) mentioned treating with antibiotics including amoxicillin/clavulanic acid or doxycycline if unable to take penicillin and a short course of vaginal 2% clindamycin cream or metronidazole. Several studies provided more general vaginitis treatment suggestions including discontinuing antifungal treatment, using only water and not soap to wash, and refraining from sexual intercourse until symptoms resolve.

**Table 5:** Summary of treatment recommendations (n = 35 studies).

		Study reference numbers	Number of studies	Percentage of studies^b^
Baking soda treatment^a^				
	Sitz bath	17, 18, 20, 34, 36, 38, 40, 42, 53, 58, 66, 70, 72, 73	14	40%
	Irrigation	7, 8, 10, 17, 18, 20, 23, 28, 30, 34, 37, 38, 40, 42, 44, 45, 46, 48, 50, 51, 52, 54, 58, 59, 60, 62, 66, 70, 72, 73	30	86%
	Capsules	46, 62	2	6%
**Other treatment**				
	Discontinuing tampons	18, 38, 40, 42, 53, 58	6	17%
	Antibiotics	17, 20, 30, 54, 74	5	14%
	Sertaconazol vaginal suppository and an alkaline (pH 8–9) vaginal moisturizer	43	1	3%
**Primary or secondary information source**				
	Primary	7, 8, 23, 27, 30, 36, 43, 50, 51, 53, 58–60, 66	14	40%
	Secondary	10, 17, 18, 20, 28, 34, 37, 38, 40, 42, 44–46, 48, 52, 54, 62, 70, 72–74	21	60%
**Type of study**				
	Review study	7, 8, 10, 17, 18, 27, 28, 30, 34, 37, 38, 40, 42, 44–46, 48, 50, 52, 54, 58, 59, 62, 66, 70	25	71%
	Cross-sectional descriptive	20, 23, 36, 51, 53, 72	6	17%
	Prospective/retrospective cohort	43, 74	2	6%
	Case series	60	1	3%
	Cross-sectional analytical	73	1	3%

^a^More than 35 treatment recommendations (the number of studies that provided treatment recommendations) as some studies recommended multiple treatments.

^b^ The percentages may not total 100% due to rounding.

### Associations between CV and other conditions

Table 6 shows the studies that examined conditions associated with CV with an assessment of bias. The details of the bias assessment are provided in S6 Table. There were 8 studies [13%] reporting associations between CV and other conditions that can be divided into 3 topics: pregnancy, cervical dysplasia, and other.

**Table 6:** Association between CV and other conditions (n = 8 studies).

Publication	Location	Number of women	Study type	Exposure/outcome	Selection bias	Information bias	Confounder bias	Results
Pregnancy								
Akgun 2012 [15] (abstract)	Turkey	4672	retrospective cohort	CV/infertility	Low	High	High	In women with CV, 32.9% women were infertile; in women without CV, 5.58% were infertile (*P* < .05).
Rocchetti 2011 [55]	Brazil	405	cross-sectional analytical	vaginal flora/group B strep colonization	Low	Moderate	Low	In women with group B strep colonization compared to not having group B strep colonization, the odds of CV was 2.717 (95% CI, 1.075–6.866).
Zidovsky 1963 [74]	Czech Republic	953	retrospective cohort	CV /fetal impairment	Moderate	Moderate	High	In women with CV, 18.0% (95% CI, 12.1%-33.9%) of infants were impaired/died; in women without CV 3.9% (95% CI, 2.7%-5.9%) of infants were impaired or died.
**Dysplasia**								
Nasiell 1972 [49]	Sweden	440	cross-sectional analytical	CV/cervical dysplasia and cervical cancer	Low	Moderate	High	In women with invasive carcinoma, 4% had CV compared to 12% in women with carcinoma in situ; 9% in women with dysplasia; and 19% in controls. (No statistical tests were done on these figures.)
Silva 2014 [61]	Brazil	3390	retrospective cohort	CV/HPV and cervical intraepithelial lesions	Low	Moderate	Moderate	Prevalence of CV among women with low-grade intraepithelial lesions (LSIL) or lesions of undetermined significance that evolved to high-grade intra-epithelial lesion (HSIL) was 3.7% (15/409), compared to a prevalence of 5.8% (175/2981) in women with lesions that did not evolve into HSIL. The study indicates that statistical testing was done but did not provide this information.
Vieira-Baptista 2017 [65] (abstract)	Portugal	1022	cross-sectional analytical	CV/HPV infection and cervical dysplasia	Low	Low	High	In women with an abnormal Pap result compared to women with a normal Pap result, the prevalence of CV was 3.5% vs 2.6%, *P* = .4. In women with HR-HPV positive compared to women HR-HPV negative, the prevalence of CV was 2.7% vs 3.5%, *P* = 0.5. In women with a cervical biopsy with high-grade lesions compared to low-grade lesions, the prevalence of CV was 4.2% vs 1.8%, *P* = .3.
**Other**								
Moghaddam 2009 [47]	Iran	415	case control	lactobacillus flora/vaginal candidiasis	High	High	High	In women with candidiasis, 9% had findings of CV compared to 25% in women without candidiasis (chi-squared test appears to have been used to analyze the results between the three types of flora and *P* reported as < .0001. However, we recalculated and *P* should be < .001).
Vieira-Baptista 2017 [64] (abstract)	Portugal	291	case control	vaginal flora/vulvodynia	Low	Low	Moderate	In women with vulvodynia compared to women without vulvodynia, the odds of CV are 4.593 (95% CI, 1.890–11.160).

Three studies looked at pregnancy and each focused on a different aspect: infertility [15], fetal impairment [74], and group B strep [55]. CV was found to be associated with an increased risk of infertility and an increased risk of fetal impairment, but both studies had a moderate-to-high risk of bias [15,74]. The study on group B strep found that women with group B strep had increased odds of also having CV and had a low-to-moderate risk of bias [55].

Three studies examined whether there was any association between cervical dysplasia and CV: two studies found no association (low-to-moderate risk of bias) [61,65], while one study found CV was associated with a lower risk of developing a high-grade intraepithelial lesion or invasive neoplasia (moderate risk of bias) [49]. The remaining two studies found greater odds of CV in women with vulvodynia (low-to-moderate risk of bias) [64] and lower odds of their having vaginal candidiasis (high risk of bias) [47].

## Discussion

This scoping review is the first systematic review to map out the literature published on CV. We uncovered more studies (64 vs. 10) than the 2020 critical appraisal [10] because we conducted a systematic literature search and our inclusion criteria included studies in foreign languages, and studies not focused on CV. Nevertheless, our scoping review had findings similar to the 2020 critical appraisal, including the need for objective criteria and the need for evidence on treatment and treatment outcomes.

Why there is a paucity of studies on CV compared to the over 7000 studies that have been published on vaginitis [10] is unclear; is it because CV is unknown to the medical community or because it is a variant of the normal vaginal microbiome? However, given that studies of CV span 3 continents, are from diverse countries, and are published in a broad spectrum of journals, it is more suggestive that CV is a true condition.

CV is not the only vaginal microbiome dysbiosis condition that is little known and understudied. Aerobic vaginitis or desquamative inflammatory vaginitis is similar to bacterial vaginosis in that it lacks lactobacilli, but dissimilar in that the vaginal microbiome is colonized predominately by aerobic bacteria rather than by anaerobic bacteria [75]. The symptoms of aerobic vaginitis include excessive vaginal discharge, pruritis, burning, and dyspareunia [75]. In addition, there is also a controversial entity characterized by the presence of abnormally long possible lactobacilli (length of 40 um-75 um instead of 5 um-15 um), referred to as leptothrix, fusiform lactobacilli, and lactobacillosis; it is found to coexist with other vaginal dysbiosis and infectious conditions as well as normal flora [66,76]. It often does not have symptoms (based on author experiences), unclear whether it is causative or incidental [77], and unknown whether it is an abnormally long *Lactobacillus* sp. or a different bacteria species [76–78].

Although the studies in this scoping review can be used to provide an estimate of the median prevalence of CV (5% [IQR 3%-8%] in women with symptoms and 22% [IQR 19%-24%] in women with recurrent symptoms), and the differences in prevalence among subgroups help provide credibility to the prevalence estimate, this estimate is limited by 1) quality of studies; 2) lack of standard criteria used to diagnose CV; and 3) insufficient number of studies overall and in subgroups. To inform clinicians whether and how much CV should be considered, further studies on prevalence using gold-standard diagnostic criteria in symptomatic women and asymptomatic women in various geographic locations are needed.

The subjective criteria used by studies in this scoping review to diagnose CV highlight the need to have a gold-standard objective criterion. There is some movement in this direction; for instance, Hu et al. found distinct differences in quantity of *Lactobacillus* spp., percentage of fragmented epithelial cells, and percentage of whole epithelial cells between women presenting with CV and other vaginosis conditions [39], whereas Hacisalihoglu et al. scored cytolysis, lactobacilli quantity, neutrophils, finding of bacterial vaginosis, *Candida* spp. hyphae/spores, and *Trichomonas vaginalis* based on quantity under oil immersion (0–3 scale) [36]. However, they reported only individual scores rather than also providing a composite score.

**Fig 3:**
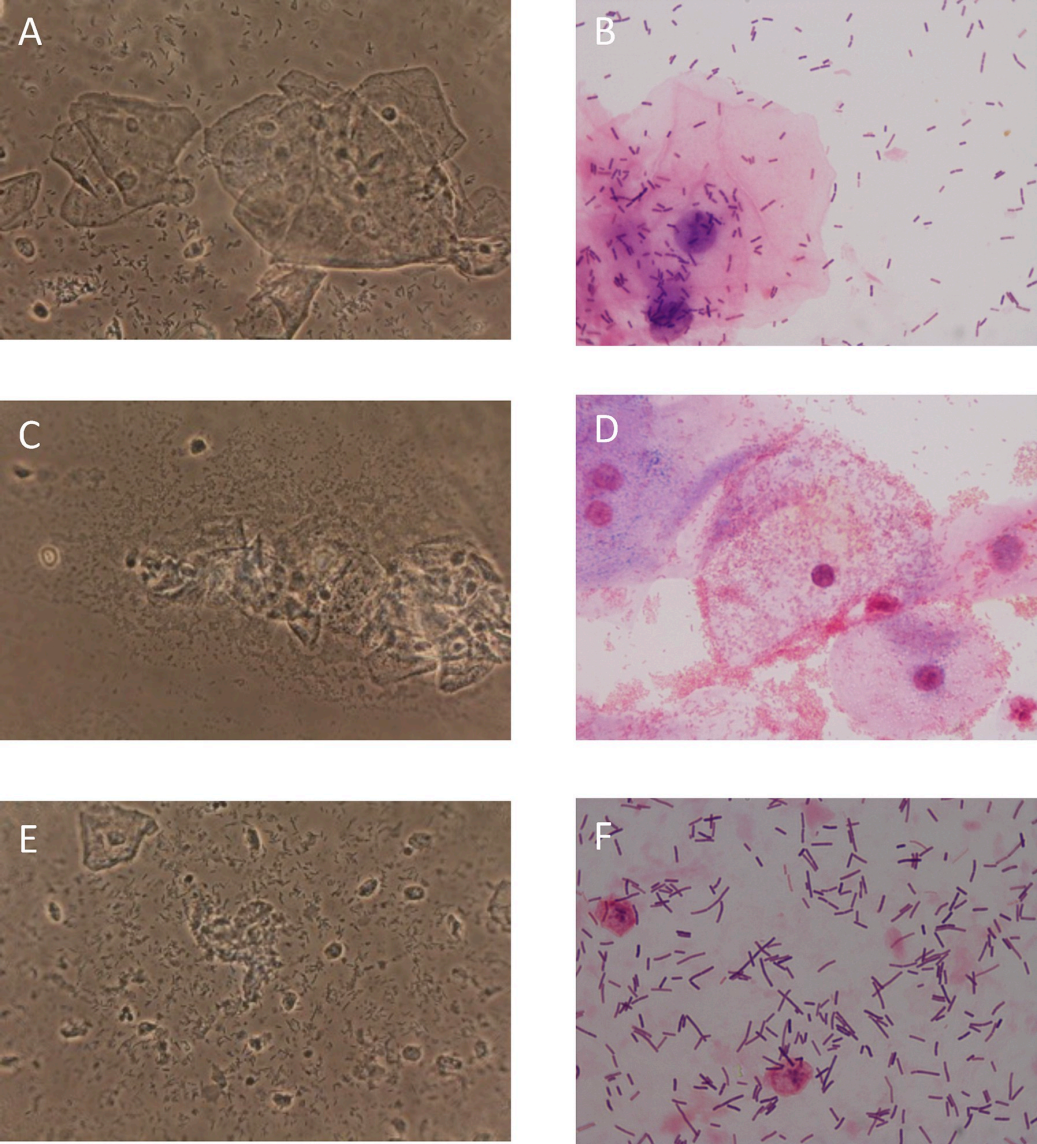
Wet mounts and gram stains. A & B, Normal wet mount and gram stain (pleomorphic lactobacilli and superficial cells). C & D, Bacterial vaginosis wet mount and gram stain (lack of lactobacilli, clue cells, and granular flora). E & F, CV wet mount and gram stain (abundant lactobacilli, fragmented epithelial cells: Bare nuclei and cytoplasmic debris). Wet mount magnification: 400x and gram stain magnification 1000x.

Perhaps summarizing the percentage of cytolysis, quantity of lactobacilli, and lack of other vaginosis findings into a composite score similar to Donder’s criteria for aerobic vaginitis [75] or Nugent’s score for bacterial vaginosis [79] may be a way forward. This should be possible using either a wet mount or a Gram stain. The wet mount is advantageous as it enables clinicians to diagnose CV quickly during a clinic visit; however, in practice, Gram stains are usually completed at the laboratory due to the lack of microscopes and expertise in clinic [68,80]. Another option may be modeling it after Hay/Ison criteria, which classify vaginal flora into grades and require less skill and time [81]. In addition, with the possible shift to molecular diagnosis including a nucleic acid amplification test [67], a gold standard may need to take this into account as well. Fig 3 shows illustrations of wet mounts and gram stains for normal, bacterial vaginosis, and CV.

The vaginal community state type is the framework often used to categorize the vaginal microbiome [82]. In our scoping review, no studies that matched the inclusion criteria explicitly examined how CV fits into this framework. However, there were studies that examined the microbiology of CV and found the *Lactobacillus crispatus* dominates which is most consistent with vaginal community state type I [83].

There were only a few studies evaluating treatment for CV, and the results infer that increasing vaginal pH with baking soda is effective. However, these studies were observational, primarily included a single exposure and outcome, had a small number of participants, and did not include microbiological results post-treatment. Studies with a more rigorous design, including randomized controlled trials, would be useful to further delineate treatment effectiveness. In addition, it would be advantageous for studies to explore more definitive treatment options. Other vaginal dysbiosis conditions (for instance, bacterial vaginosis) are treated with antibiotics or antiseptics with a curative intent [66].

Some studies examined associations between CV and other conditions such as cervical dysplasia and pregnancy/fertility. However, these studies have, on average, a moderate risk of bias and there are few such studies, so it is difficult to make any inferences. There was an additional prospective cohort study on pregnancy outcomes of 2453 women by Bercovici et al. [84] in 1973 that found cytolysis increased from first to second to third trimester before decreasing prior to delivery; the incidence of cytolysis was significantly higher in women with hyperemesis gravidarum and diabetes and did not appear to have any adverse fetal outcomes. However, the study did not consider the quantity of lactobacilli [84].

It is possible that we missed capturing studies on CV as we did not examine gray literature and only reviewed citations of studies that focused on CV. However, given that our scoping review included more studies than previous reviews, it is unlikely that any potentially missed studies would significantly impact our results. Due to limited resources, we only assessed bias of studies that focused on conditions associated with CV, as it was most important to determine bias for these studies. Our review of treatment included primary and secondary sources and as such, it is possible that some information was repetitive; however, secondary sources were included because it was difficult to discern whether authors’ experiences with treatment were included in studies that referenced treatment recommendations.

## Conclusion

This scoping review clearly shows that there is a lack of robust evidence along all aspects of CV. Historically, CV has been discounted based on lack of evidence, and its symptoms have been explained as simply physiological or even psychological. However, we feel that it is important to consider CV, given the volume of consistent evidence supporting this condition from a diverse range of countries and sources, and the potential for distressing symptoms if left untreated. Future research should especially be centered around establishing gold-standard diagnostic criteria that will enable practitioners, laboratories, and researchers to better characterize, diagnose, and confirm the validity of this equivocal condition.

## Supporting information

S1 Table Vaginitis differential. (PDF)

S2 Table PRISMA-ScR checklist. (PDF)

S3 Table PRISMA-S checklist. (PDF)

S4 Table Full data set. (XLSX)

S5 Table Likelihood ratio calculation for Yang et al.’s study. (PDF)

S6 Table Bias assessment of studies on the association between cytolytic vaginosis and other conditions. (PDF)

S1 File Search strategies. (PDF)
