# Supplementary material for: Scoping review of cytolytic vaginosis literature
Source: PLoS One. 2023 Jan 26;18(1):e0280954. doi: 10.1371/journal.pone.0280954 (PMC9879469; doi:10.1371/journal.pone.0280954)
Supplement: S1 Table — (PDF) [file pone.0280954.s001.pdf]

Scoping Review of Cytolytic Vaginosis Literature

S1 Table: Vaginitis differential diagnosis

	More common	Less common
Dermatologic	Contact dermatitis (allergic and irritant), lichens simplex chronicus, psoriasis, atopic dermatitis, folliculitis, lichen sclerosus, atrophic vaginitis	Lichens planus, hidradenitis suppurativa, pityriasis rosea, prurigo nodularis, and autoimmune blistering diseases
Infectious	Candidiasis, trichomonas, herpes simplex, gonorrhea, chlamydia	Tinea cruris and versicolor, impetigo, perianal streptococcal infection, and scabies
Vaginal dysbiosis	Bacterial vaginosis	Cytolytic vaginosis, aerobic vaginitis/desquamative inflammatory vaginitis, leptothrix
Malignancy/premalignancy		Cervical cancer (squamous cell carcinoma and adenocarcinoma), HSIL (high grade squamous, intraepithelial lesion), Vulvar cancer (squamous cell carcinoma, verrucous carcinoma, basal cell carcinoma, and sarcoma), dVIN (differentiated vulvar intraepithelial neoplasia), Extramammary Paget's disease
Other		Crohn's disease, insect bites, and trauma
