# Supplementary material for: Scoping review of cytolytic vaginosis literature
Source: PLoS One. 2023 Jan 26;18(1):e0280954. doi: 10.1371/journal.pone.0280954 (PMC9879469; doi:10.1371/journal.pone.0280954)
Supplement: S5 Table — (PDF) [file pone.0280954.s005.pdf]

Scoping Review of Cytolytic Vaginosis Literature

S5 Table: Liklihood ratio of symptoms/signs in Yang 2017 study

	Sensitivity	Specificity	Positive predictive value	Negative predictive value	Positive liklihood ratio	Negative liklihood ratio
Discharge - paste like	60%	71%	61%	71%	**2.10**	**0.56**
Discharge - large quantity	44%	72%	54%	64%	**1.60**	**0.77**
Symptoms - luteal phase	80%	38%	49%	72%	**1.29**	**0.53**
Signs - no swelling	52%	67%	54%	66%	**1.58**	**0.71**
Signs - no erosions	68%	57%	54%	71%	**1.58**	**0.56**
Signs - no ulcerations	85%	42%	52%	79%	**1.47**	**0.36**
