# Supplementary material for: Scoping review of cytolytic vaginosis literature
Source: PLoS One. 2023 Jan 26;18(1):e0280954. doi: 10.1371/journal.pone.0280954 (PMC9879469; doi:10.1371/journal.pone.0280954)
Supplement: S6 Table — (PDF) [file pone.0280954.s006.pdf]

Scoping Review of Cytolytic Vaginosis Literature

S6 Table - Bias assessment of studies that focused on the association between cytolytic vaginosis and other conditions

Cross-sectional/cohort studies

	Other	Selection bias					Information bias									Confounders	
	1	2	3	4	5	bias	6	7	8	9	10	11	12	13	bias	14	bias
Akgun 2012 (abstract)	yes	yes	yes	yes	CD	**low**	CD	CD	NA	CD	no	CD	yes	yes	**high**	no	**high**
Nasiell 1972	yes	yes	yes	yes	no	**low**	no	no	NA	yes	no	yes	CD	yes	**mod**	no	**high**
Rocchetti 2011	yes	yes	yes	yes	no	**low**	no	no	yes	CD	no	yes	CD	yes	**mod**	yes	**low**
Silva 2014	yes	yes	yes	yes	no	**low**	yes	CD	yes	no	no	yes	CD	no	**mod**	no	**mod**
Vieira-Baptista 2017 (abstract)*	yes	yes	yes	yes	no	**low**	no	yes	NA	yes	yes	yes	yes	yes	**low**	no	**high**
Zidovsky 1963	no	no	yes	yes	no	**mod**	yes	yes	NA	yes	no	CD	CD	yes	**mod**	no	**high**

CD: cannot determine
NA: not applicable
*information from abstract and communication with author

National Institute of Health Tool bias assessment questions: https://www.nhlbi.nih.gov/health-topics/study-quality-assessment-tools
1. Was the research question or objective in this paper clearly stated?
2. Was the study population clearly specified and defined?
3. Was the participation rate of eligible persons at least 50%?
4. Were all the subjects selected or recruited from the same or similar populations (including the same time period)? Were inclusion and exclusion criteria for being in the study prespecified and applied uniformly to all participants?
5. Was a sample size justification, power description, or variance and effect estimates provided?
6. For the analyses in this paper, were the exposure(s) of interest measured prior to the outcome(s) being measured?
7. Was the timeframe sufficient so that one could reasonably expect to see an association between exposure and outcome if it existed?
8. For exposures that can vary in amount or level, did the study examine different levels of the exposure as related to the
9. Were the exposure measures (independent variables) clearly defined, valid, reliable, and implemented consistently across all study participants?
10. Was the exposure(s) assessed more than once over time?
11. Were the outcome measures (dependent variables) clearly defined, valid, reliable, and implemented consistently across all study participants?
12. Were the outcome assessors blinded to the exposure status of participants?
13. Was loss to follow-up after baseline 20% or less?
14. Were key potential confounding variables measured and adjusted statistically for their impact on the relationship between exposure(s) and outcome(s)?

Case-control studies

	Other	Selection bias							Information bias				Confounders	
	1	2	3	4	5	6	7	8	bias	9	10	11	bias	12	bias
Moghaddam 2009	yes	no	no	CD	no	yes	CD	no	**high**	no	yes	no	**high**	no	**high**
Vieira-Baptista 2017 (abstract)*	yes	yes	no	yes	yes	yes	NA	no	**low**	no	yes	yes	**low**	yes	**mod**

CD: cannot determine
NA: not applicable
*information from abstract and communication with author

National Institute of Health Tool bias assessment questions: https://www.nhlbi.nih.gov/health-topics/study-quality-assessment-tools
1. Was the research question or objective in this paper clearly stated and appropriate?

2. Was the study population clearly specified and defined?
3. Did the authors include a sample size justification
4. Were controls selected or recruited from the same or similar populations that gave rise to the cases (including the same timeframe)?
5. Was the definitions, inclusion and exclusion criteria, algorithms or processes used to identify or select cases and controls valid, reliable and implemented consistently across all study participants?
6. Were the cases clearly defined and differentiated from controls?
7. If less than 100 percent of eligible cases and/or controls were selected for the study, were the cases and/or controls randomly selected from those eligible?
8. Was there use of concurrent controls?
9. Were the investigators able to confirm that the exposure/risk occurred prior to the development of the condition or event that defined a participant as a case?
10. Were the measures of exposure/risk clearly defined, valid, reliable, and implemented consistently (including the same time period) across all study participants?
11. Were the assessors of exposure/risk blinded to the case or control status of participants?
12. Were key potential confounding variables measured and adjusted statistically in the analysis? If matching was used, did the investigators account for matching during study analysis?
