# Supplementary material for: Scoping review of cytolytic vaginosis literature
Source: PLoS One. 2023 Jan 26;18(1):e0280954. doi: 10.1371/journal.pone.0280954 (PMC9879469; doi:10.1371/journal.pone.0280954)
Supplement: S1 File — (PDF) [file pone.0280954.s007.pdf]

**Scoping Review of Cytolytic Vaginosis Literature**
**S1 File – Search Strategy**

## Ovid MEDLINE(R) and Epub Ahead of Print, In-Process & Other Non-Indexed Citations and Daily <1946 to April 03, 2019>

| # | Search Statement | Results |
|---|---|---|
| 1 | (cytolytic vaginosis or "lactobacillus overgrowth").mp. | 25 |
| 2 | lactobacillus.mp. or exp Lactobacillus/ or Doderlein*.mp. [mp=title, abstract, original title, name of substance word, subject heading word, floating sub-heading word, keyword heading word, organism supplementary concept word, protocol supplementary concept word, rare disease supplementary concept word, unique identifier, synonyms] | 36064 |
| 3 | (cytoly* or lysis).mp. [mp=title, abstract, original title, name of substance word, subject heading word, floating sub-heading word, keyword heading word, organism supplementary concept word, protocol supplementary concept word, rare disease supplementary concept word, unique identifier, synonyms] | 60555 |
| 4 | 2 and 3 | 245 |
| 5 | exp Vagina/ or exp Vaginal Diseases/ or vagin*.mp. | 145852 |
| 6 | 4 and 5 | 29 |
| 7 | 1 or 6 | 42 |
| 8 | remove duplicates from 7 | 42 |

## Embase <1974 to 2019 April 03>

| # | Search Statement | Results |
|---|---|---|
| 1 | (cytolytic vaginosis or "lactobacillus overgrowth").mp. | 32 |
| 2 | lactobacillus.mp. or exp Lactobacillus/ or Doderlein*.mp. [mp=title, abstract, heading word, drug trade name, original title, device manufacturer, drug manufacturer, device trade name, keyword, floating subheading word, candidate term word] | 43670 |
| 3 | (cytoly* or lysis).mp. [mp=title, abstract, heading word, drug trade name, original title, device manufacturer, drug manufacturer, device trade name, keyword, floating subheading word, candidate term word] | 119883 |
| 4 | 2 and 3 | 323 |

| | | |
|---|---|---|
| 5 | exp vagina/ or exp vagina disease/ or vagin*.mp. | 20372 7 |
| 6 | 4 and 5 | 44 |
| 7 | 1 or 6 | 60 |
| 8 | remove duplicates from 7 | 58 |

**PROSPERO Searched April 43, 2019 Results =0**

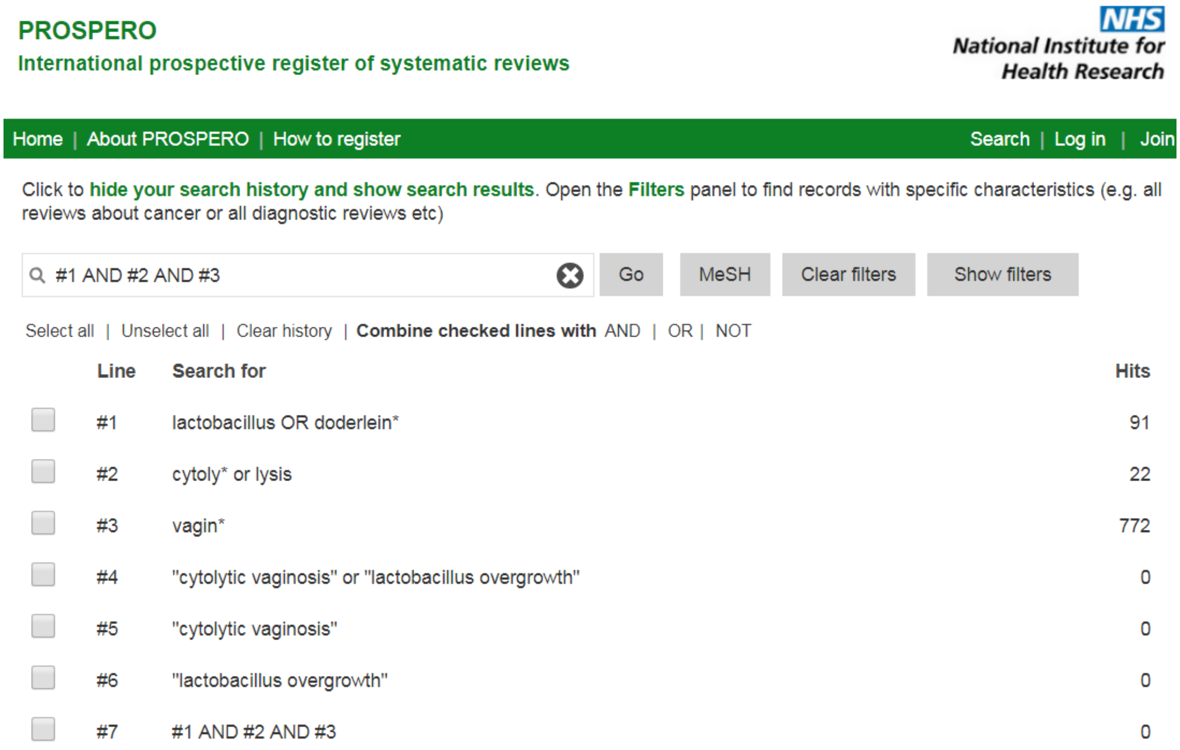

PROSPERO

International prospective register of systematic reviews

NHS National Institute for Health Research

Home | About PROSPERO | How to register Search | Log in | Join

Click to **hide your search history and show search results**. Open the **Filters** panel to find records with specific characteristics (e.g. all reviews about cancer or all diagnostic reviews etc)

#1 AND #2 AND #3 Go MeSH Clear filters Show filters

Select all | Unselect all | Clear history | **Combine checked lines with** AND | OR | NOT

| Line | Search for | Hits |
|---|---|---|
| #1 | lactobacillus OR doderlein* | 91 |
| #2 | cytoly* or lysis | 22 |
| #3 | vagin* | 772 |
| #4 | "cytolytic vaginosis" or "lactobacillus overgrowth" | 0 |
| #5 | "cytolytic vaginosis" | 0 |
| #6 | "lactobacillus overgrowth" | 0 |
| #7 | #1 AND #2 AND #3 | 0 |

**CINAHL Searched April 4, 2019 Results =9**

### Search History/Alerts

Print Search History    Retrieve Searches    Retrieve Alerts    Save Searches / Alerts

Select / deselect all | **Search with AND** | **Search with OR** | **Delete Searches**

| Search ID# | Search Terms | Search Options | Actions |
|---|---|---|---|
| S1 | (MH "Lactobacillus+") OR "lactobacillus" or Doderlein* | **Search modes** - Find all my search terms | **View Results** (2,999 |
| S2 | cytoly* or lysis | **Search modes** - Find all my search terms | **View Results** (2,073 |
| S3 | (MH "Vagina") OR "vagina" OR (MH "Vaginal Diseases+") | **Search modes** - Find all my search terms | **View Results** (8,891 |
| S4 | S1 AND S2 AND S3 | **Search modes** - Find all my search terms | **View Results** (3) |
| S5 | "cytolytic vaginosis" or "lactobacillus overgrowth" | **Search modes** - Find all my search terms | **View Results** (9) |
| S6 | S4 OR S5 | **Search modes** - Find all my search terms | **View Results** (9) |

## SCOPUS Searched April 4, 2019 Results = 61

( TITLE-ABS-KEY ( ( lactobacillus OR doderlein* ) AND ( cytoly* OR lysis ) AND vagin* ) OR TITLE-ABS-KEY ( cytolytic AND vaginosis OR "lactobacillus overgrowth" ) )

## Cochrane Library Searched April 4, 2019 Results =61

| Cochrane Reviews | Cochrane Protocols | Trials | Editorials | Special collections | Clinical Answers | Other Reviews |
|---|---|---|---|---|---|---|
| 0 | 0 | 0 | 0 | 0 | 0 | |

**0** Cochrane Reviews matching on **( lactobacillus OR doderlein* ) AND ( cytoly* OR lysis ) AND vagin* in Title Abstract Keyword OR "cytolytic vaginosis" OR "lactobacillus overgrowth" in Title Abstract Keyword - (Word variations have been searched)**

**Cochrane Database of Systematic Reviews**
Issue 4 of 12, April 2019

## ProQuest Dissertations & Theses Global Searched April 4, 2019 Results =2

noft(( ( lactobacillus OR doderlein* ) AND ( cytoly* OR lysis ) AND vagin* ) ) OR noft("cytolytic vaginosis" OR "lactobacillus overgrowth" )
